# Prospects for breast cancer immunotherapy using microRNAs and transposable elements as objects

**DOI:** 10.37349/etat.2024.00261

**Published:** 2024-08-06

**Authors:** Rustam Nailevich Mustafin

**Affiliations:** Hospital del Mar Medical Research Institute, Spain; Department of Medical Genetics and Fundamental Medicine, Bashkir State Medical University, Ministry of Health of Russia, 450008 Ufa, Russia

**Keywords:** Immunotherapy, inhibitors, microRNA, reverse transcriptase, breast cancer, retroelements, transposons

## Abstract

One of the directions in treatment of chemoresistant breast cancer (BC) may include new methods of activating the immune response against tumor cells. Clinically used checkpoint inhibition using antibodies to PD-1 and PD-L1 works in some patients, but the lack of biomarkers means number of respondents is low. The possibility of combining this method with chemotherapy is limited by an increased risk of toxic liver damage, development of immune-related pneumonitis, and thyroid dysfunction. This article includes introduction into the clinic of new methods of immunotherapy for BC, among which epigenetic activation of retroelements, double-stranded transcripts of which stimulate the interferon response against the tumor, is promising. For this purpose, inhibitors of DNA methyltransferase*, histone deacetylase* and histone methyltransferase* are used (* subtitles in the main text). Their antitumor effect is also mediated by removal of repressive epigenetic marks from tumor suppressor genes. However, numerous studies have proven the role of retroelements in the carcinogenesis of various malignant neoplasms, including BC. Moreover, endogenous retroviruses HERV-K and LINE1 retrotransposons are planned to be used as diagnostic biomarkers for BC. Therefore, a rational approach to using viral mimicry in antitumor therapy of BC may be the simultaneous suppression of specific retrotransposons (drivers for carcinogenesis) using reverse transcriptase inhibitors and silencing of specific transposons involved in carcinogenesis using complementary microRNAs. To determine possible pathways of influence in this direction, 35 specific transposon-derived microRNAs* changes in BC were identified, which can become guides for targeted therapy of BC.

## Introduction

Breast cancer (BC) is a heterogeneous disease with different pathological and clinical features, according to which it is characterized by a pronounced diversity of response to therapy [1]. According to the International Agency for Research on Cancer (https://gco.iarc.fr/today/), the incidence of BC in the world for 2020, regardless of gender, is 58.5 per 100,000 population, which exceeds these figures for all other cancers. In Russia, BC also ranks first in terms of prevalence and incidence, regardless of gender (11.8% of all cancers) [2]. According to immunohistochemical markers, BC is classified into luminal-A, luminal-B HER2-negative, luminal-B HER2-positive, HER2-enriched and triple-negative BC (TNBC) [1]. BC that expresses progesterone receptors (PR) or estrogen receptors (ER) is called luminal BC. For the treatment of BC, surgical removal of the tumor with maximum preservation of healthy tissue, radiation, hormonal (for BC with PR and ER expression) and chemotherapy (a combination of antimetabolites, anthracycline and cylophosphamide) are used [3].

A promising direction in the treatment of chemoresistant BC are immunotherapy methods, the most famous of which is inhibition of checkpoints (negative regulatory pathways by which cancer cells avoid detection by the immune system). Checkpoints for programmed cell death (PD-1—programmed cell death 1) and cytotoxic T-lymphocyte protein (CTLA4—cytotoxic T-lymphocyte protein 4) are known. The cell surface receptor PD-1 is expressed by T cells when activated during priming or expansion and binds to one of two ligands: programmed cell death ligand 1 (PD-L1) and PD-L2, which are produced by normal and tumor cells in response to cytokines (such as interferon-γ). When PD-L1 or PD-L2 binds to PD-1 receptors, a signal is generated that inhibits the activity of T lymphocytes. CTLA4 is also a negative regulator of T cells, controlling their activation by competing with the co-stimulatory molecule CD28 for binding to the common ligands CD80 and CD86. Antibodies that block the interaction of PD-L1 with PD-1 have an antitumor effect due to the activation of the immune response to cancer cells [4]. In clinical practice, antibodies to α-PD-1 (anti-PD-1), such as nivolumab, pembrolizumab, cemiplimab, sintilimab, camrelizumab, toripalimab, tislelizumab, zimberelimab, prolgolimab, and dostarlimab, have been successfully used in cancer treatment. In addition, antibodies to α-PD-L1 (anti-PD-L1) are used: atezolizumab, durvalumab, and avelumab [5]. However, the results of clinical studies indicate the low effectiveness of checkpoint therapy in BCs, which is associated with the peculiarities of expression of PD-1 and PD-L1 by these tumors. Thus, in a study of 3,916 BC samples, the production of PD-L1 by cells of the immune system was determined in 6%, and by tumor cells in 1.7% of biopsies with pronounced differences depending on the type of tumor (19% for the basal-like subtype of BC) [6]. In other studies, an analysis of 650 BC tissues revealed the expression of PD-L1 in 23.4% [7], a study of 5,454 BC samples determined the expression of PD-L1 in 20% of cases (with the basal-like subtype—in 38%) [8]. TNBC has the greatest immunogenicity, and therefore clinical studies of anti-PD-1/PD-L1 were conducted for this type of tumor, the response to which was determined in 18.5 [9]—20% of patients [10]. In patients with luminal BC, only a partial response to anti-PD-L1 was detected in 12% of patients [11].

However, clinical trial results are emerging showing conflicting results about effectiveness of anti-PD-L1 in BC treatment. In 2018, the results of a phase 1b JAVELIN Solid Tumor study using avelumab, an anti-PD-L1 antibody, in patients with locally advanced and metastatic BC were published. Avelumab showed an acceptable safety profile and clinical activity in a subset of patients with metastatic BC. The confirmed objective response rate (ORR) was 3.0% overall and 5.2% in patients with TNBC. A trend toward a higher ORR was seen in patients with PD-L1^+^ vs. PD-L1^–^ tumor-associated immune cells in the overall population (16.7% vs. 1.6%) and in the TNBC subgroup (22.2% vs. 2.6%) [12]. A pilot clinical trial of immuno-priming durvalumab with bevacizumab in HER2-negative advanced BC, conducted in 2020, showed encouraging activity in a heavily pre-treated cohort. Clinical benefit rate at 2 months and 4 months was 60% and 44%, respectively, without significant differences between hormone-positive and triple-negative [13].

In 2021, the results of an open-label, randomized, phase 3 trial Chemotherapy with or without avelumab followed by avelumab maintenance vs. alone chemotherapy in patients with previously untreated epithelial ovarian cancer (JAVELIN Ovarian 100) were published. Results did not support the use of avelumab in the frontline treatment setting. Moreover, serious adverse events of any grade occurred in 28% patients in the avelumab maintenance group, 136% in the avelumab combination group, and 19% in the control group [14]. In 2022, the randomized, double-blind phase 2b ALICE trial of atezolizumab plus anthracycline-based chemotherapy in metastatic TNBC was conducted. The progression-free proportion after 15 months was 14.7% in the atezo-chemo arm vs. 0% in the placebo-chemo arm. Adverse events leading to drug discontinuation occurred in 18% of patients in the atezo-chemo arm and in 7% of patients in the placebo-chemo arm [15]. In 2023, the randomized SYNERGY phase I/II trial study of the role of oleclumab in combination treatment with anti-PD-L1 durvalumab and carboplatin showed that the 24-week clinical benefit rate was not significantly improved by adding oleclumab [16]. In 2024, the results of a multicenter phase II trial of the anti-PD-L1/CTLA4 bispecific antibody KN046 in combination with nab-paclitaxel in first-line treatment of metastatic TNBC were published. In general, KN046 combined with nab-paclitaxel showed favorable efficacy and survival benefits with tolerable toxicity in the first-line treatment of metastatic TNBC, especially PD-L1 positive [17].

In addition, meta-analyses and clinical trials have shown that the use of PD-L1 and PD-1 inhibitors significantly increases the risk of liver toxicity [18], immune-related pneumonitis [19], thyroid dysfunction (especially hypothyroidism) [20], pruritus, fatigue, rash, and nausea (especially with combinations of anti-PD-L1 and anti-PD-1) [21]. Therefore, it is necessary to introduce into the clinic new methods of immunotherapy for BC, among which the activation of antitumor immunity by double-stranded RNAs, which are formed through the artificial activation of retroelements (REs), is promising. For this purpose, an inhibitor of the protein kinase WEE1 can be used (normally it stops the division of cells with damaged DNA), which enhances the expression of endogenous retroviruses (ERVs), the double-stranded transcripts of which stimulate the interferon response to cancer [22]. The most promising is the activation of REs by influencing epigenetic factors, the effect of which is reversible and allows for a differentiated approach with the possibility of inhibiting specific REs involved in the pathogenesis of BC.

## Viral mimicry method in the treatment of BC

Epigenetic factors include DNA methylation, histone modifications, and RNA interference by noncoding RNAs. All of them are interconnected and provide dynamic regulation of genes and REs expression during organism development and aging [23]. REs are transposable elements that are mobilized through a copy-and-paste mechanism. Transposable elements also include a class of DNA transposons that are mobilized through a cut-and-paste mechanism. REs include non-long terminal repeat (non-LTR) autonomous long interspersed nuclear element (LINE) elements and non-autonomous short interspersed nuclear element (SINE; including Alu) and SINE-VNTR-Alu retroelement (SVA) elements. LTR-containing REs include ERVs [1]. REs occupy a significant part of the human genome: LINE1 account for 17% of all DNA sequences [24], human endogenous retroviruses (HERV)—8% [25], Alu—10% [26]. Despite this, REs expression promotes activation of the antiviral interferon response to double-stranded REs transcripts [22], which is the cause of aging and aging-associated diseases [23]. This property is used in the method of viral mimicry, in which the expression of REs is stimulated using inhibitors of DNA methyltransferases, histone deacetylases and histone methyltransferases (Table 1). The method of viral mimicry is based on the activation with the help of drugs in cancer of REs, the transcription and translation products of which are similar in nature (due to their origin in evolution from viruses) to viruses, and therefore stimulate an immune response against cancer. As a result, the cancer is attacked by immune cells and destroyed. An example is 5-aza-2-deoxycytidine, which utilizes a demethylation mechanism and has been approved for use in cancer treatment by the US Food and Drug Administration [27]. In addition to the attack of cancer by immune cells, which is associated with antiviral interferon response to double-stranded REs transcripts [22], drugs used for viral mimicry have other additional antitumor effects. For example, 5-aza-2-deoxycytidine induces differentiation, apoptosis, and senescence in cancer cells due to regulatory effects on the expression of various genes. This is due to the effect of demethylation on the activation of the expression of not only REs, but also tumor suppressor genes that suppress the growth of BC, such as BRCA1, BRCA2, p15, and p16 [27]. In the method of viral mimicry, Guadecitabine upregulates MHC-I in tumor cells promoting recruitment of CD8^+^ T cells to the microenvironment. *MHC-I* genes are upregulated in BC patients treated with hypomethylating agents [22].

**Table 1:** Methods of viral mimicry for the treatment of BC

**Drug categories**	**Drug names**	**Methods for assessing the effects of drugs [Reference]**
DNA methyltransferase inhibitors*	5-aza-2-deoxycytidine, guadecitabine	Human BC stem cells [27]
	Guadecitabine	Experiments on mice, clinical trials [28, 29]
	Decitabine	Human BC cell line and experiments on mice [31]
	CC-486 + durvalumab (immune checkpoint inhibitor)	Clinical trials (phase II) [32]
Bifunctional DNA methyltransferase and histone deacetylase inhibitors*	J208	TNBC cell line [33]
Histone deacetylase inhibitors*	Depsipeptide, trichostatin A	BC cell line [34, 35]
	Didemnin B (cyclic depsipeptide)	Clinical trials (phase II) [36]
	15a	Human BC cell line [37]
	12a-c	TNBC cell line [38]
	Vorinostat	Basal-like BC cell line [39]
		Clinical trials (phase IB) [40]
	Entinostat	Clinical trials (phase II [41], phase III [42])
	Panobinostat	TNBC cell line [49]
Histone methyltransferase inhibitors*	EPZ004777	Antiestrogen-resistant BC cells [43]
	EPZ-5676	TNBC stem cells [44]
	Tazemetostat, GSK126, U3i	TNBC cell line [45]

The antitumor efficacy of the DNA methyltransferase inhibitors 5-aza-2-deoxycytidine [27] and guadecitabine has been demonstrated in human BC stem cell lines [28, 29]. In mice with modeled BC, the ability of guadecitabine to enhance the expression of major histocompatibility complex MHC-I genes in response to interferon production was revealed, with the recruitment of CD8^+^ T lymphocytes into the tumor microenvironment [28]. In addition to stimulating antitumor immunity, the ability of 5-aza-2-deoxycytidine to enhance the effect of paclitaxel, adriamycin and 5-fluorouracil was discovered on BC cells [30], and low doses of deoxycytidine stimulate the expression of tumor suppressors p53, p15, p16, BRCA1, and BRCA2 [27]. Due to this, the impaired antitumor protection of BC cells is restored. In vitro on BC cells and in vivo on mice, it was shown that decitabine causes a reversion of methylation of the *PRKD1* gene promoter, blocking tumor growth and metastasis. The *PRKD1* gene is a tumor suppressor gene and encodes protein kinase D1, which prevents epithelial-mesenchymal transition of normal breast cells [31]. In clinical practice, an open-label, phase II multicohort study of an oral DNA hypomethylating agent CC-486 and durvalumab (immune checkpoint inhibitor) in BC was conducted. But the lack of robust tumor DNA demethylation was accompanied by an absence of the expected “viral mimicry” inflammatory response, and consequently, no clinical responses were observed [32].

A bifunctional histone deacetylase and DNA methyltransferase inhibitor called J208 was shown to be effective in the TNBC cell line. This drug induced apoptosis and cell cycle arrest, suppressing the migration and invasion of tumor cells [33]. Combination of 5-aza-2-deoxycytidine with the histone deacetylase inhibitor depsipeptide showed a synergistic antitumor effect against BC cells [34]. The histone deacetylase inhibitors depsipeptide and trichostatin A (TSA) also have an effect when used as monotherapy on BC cell lines [35]. But phase II clinical and pharmacological study of didemnin B in patients with metastatic BC showed no complete responses and two minor responses [36].

A new class of histone deacetylase inhibitors was designed based on the structure of flavones and isoflavones, of which lead compound 15a had a strong antiproliferative effect on SAHA-resistant BC cells. Drug 15a caused cell cycle arrest and apoptosis of BC cells [37]. Despite its activating effect on gene expression, the histone deacetylase inhibitor 12a-c was found to selectively suppress the activity of the oncogenic factor STAT3 in TNBC cell lines [38]. In basal-like BC cell lines, histone deacetylase inhibitors suberoylanilide hydroxamic acid (vorinostat or SAHA), and TSA, increased KLF5 acetylation at lysine 369 (K369), downregulated KLF5 protein expression levels, and decreased cell viability in basal-like BC cell lines [39]. A phase IB clinical trial showed the effectiveness of vorinostat in combination with ixabepilone against metastatic BC [40]. The randomized phase II clinical study, ENCORE301, evaluated the safety and efficacy of steroidal aromatase inhibitor (AI) plus entinostat (selective class I histone deacetylase inhibitor) in patients with advanced HR+ and HER2− BC [41]. In phase III clinical trials, entinostat was effective in combination with exemestane against hormone receptor-positive advanced BC [42].

H3K79 methyltransferase DOT1L is a cofactor of ERα in BC cells. Therefore, blockade of DOT1L with the drug EPZ004777 (EPZ) suppresses the proliferation of hormone-sensitive BC cells in in vitro and in vivo experiments [43]. A DOT1L inhibitor called EPZ-5676 has been shown to be effective in vitro and in vivo against TNBC stem cells [44]. For the treatment of TNBC, the use of the histone methyltransferase inhibitor tazemetostat and GSK126, which affects histone methyltransferases EZH2, is promising. In recent studies, a more specific U3i molecule of the optimized inhibitor of histone methyltransferases PROTAC (proteolytic targeting chimeras) was synthesized, which induced apoptosis of TNBC cells with minimal damage to normal cells [45].

A differentiated approach is required both in the activation of specific REs and in the selection of epigenetic factors as targets. For example, histone methyltransferase SETDB1 (synonyms EST and KMT1E), which causes silencing of REs and the *TP53* gene, has oncogenic properties, so its inhibition, in addition to the effect of viral mimicry, can have an antitumor effect by removing repressive marks of the *TP53* gene, which is a promising strategy for BC treatment [46]. At the same time, the use of 5-aza-2-deoxyuridine on BC cells increased the expression of the *Sipa1* gene, which promotes epithelial-mesenchymal transition, increasing the risk of metastases [47]. In preclinical models, it was found that histone deacetylase inhibitors increase H3K9 acetylation of the *NEDD9* gene promoter, which leads to FAK phosphorylation and aggravates BC metastasis [48]. However, the discovery of such specific pathways that impair the effectiveness of epigenetic modulators in the treatment of BC may provide the basis for combinations with other drugs that target these pathways. For example, the histone deacetylase inhibitor panobinostat on claudin-low TNBC cells markedly enhanced expression of HER3, which interacted with EGFR to activate Akt signaling. However, combination with the drug gefitinib, which inhibits HER3, can effectively stimulate apoptosis and suppress BC cell proliferation [49].

It should be noted that the problem of viral mimicry is that in this strategy increased expression of REs itself in the method of viral mimicry is a factor stimulating carcinogenesis, since a number of studies have proven the role of REs in the initiation and maintenance of the development of BC [24, 50–55]. An option for differentiated effects on specific REs in BC may be the use of monoclonal antibodies against the HERV-K envelope (env) protein, which have shown their effectiveness in experiments on mice [56]. Since microRNAs (miRNAs, especially those derived from transposable elements) cause the silencing of specific REs [57], one of the promising solutions to the problem of activation of carcinogenesis-inducing REs may be differential silencing of REs involved in the development of BC. For this purpose, it is possible to use the MDTE DB database on the origin of miRNAs from transposable elements [58], as well as use information about changes in the expression of specific RNAs in BC in the OncomiR database [59]. Among those identified through analysis of these databases, miRNAs with reduced expression (or their mimetics) can be used to suppress the activity of REs involved in BC carcinogenesis. miRNAs, the expression of which is increased in BC, are oncogenic, therefore, in the complex treatment of BC, the use of antisense oligonucleotides to suppress their activity is promising. It is also necessary to consider in more detail the role of transposable elements in the development of BC to determine more effective strategies for influencing this mechanism of BC pathogenesis.

## The role of transposable elements in the development of BC

REs are characterized by interactions with tumor suppressor genes, since the latter negatively regulate the expression of REs and contain insertional mutagenesis hotspots in their genes [23]. Activation of REs plays an important role in cancer initiation and progression by promoting oncogene expression through REs promoters. This mechanism of carcinogenesis was called onco-exaptation of REs [60] and was confirmed by meta-analyses on the significant activation of LINE [61] and Alu elements [62] in cancer tissues. Insertional mutagenesis under the influence of REs stimulates genomic instability, promoting tumor progression and clonal evolution [23]. For example, in a study of 2,954 samples of various types of cancer, transposon insertions, mainly LINE1, were identified in 50% of the BC samples [63]. Increased expression of REs with activation of 106 oncogenes was observed in 3,864 of 7,769 cancers samples. At the same time, onco-exaptation of REs was determined for half of the BC samples [60].

Analysis of HERV expression in BC tissues using reverse transcriptase PCR back in 2001 showed the presence of HERV-K transcripts (and the absence of HERV3, HERV-E4-1) in the majority of BC samples and cell lines. Full-length proviruses and mature HERV env mRNAs were detected. These changes were not found in normal breast tissue [50]. Further studies showed that the expression level of HERV-K in BC cell lines increases 5–10 times when exposed to estradiol and progesterone [51], which may indicate the role of REs in the progression of hormone-dependent BC. Indeed, suppression of HERV-K blocks the synthesis of oncogenes Ras, p-RSK, p-ERK that affect BC, and restoration of overexpression of HERV-K restores the oncogenic pathways Ras/Raf/MEK/ERK. HERV-K also activate CDK5, which suppresses the tumor suppressor p53 by impairing p53 phosphorylation. In turn, CDK5 is a mediator of TGF-β1-induced epithelial-mesenchymal transition and tumor cell migration [52].

**Figure 1:**
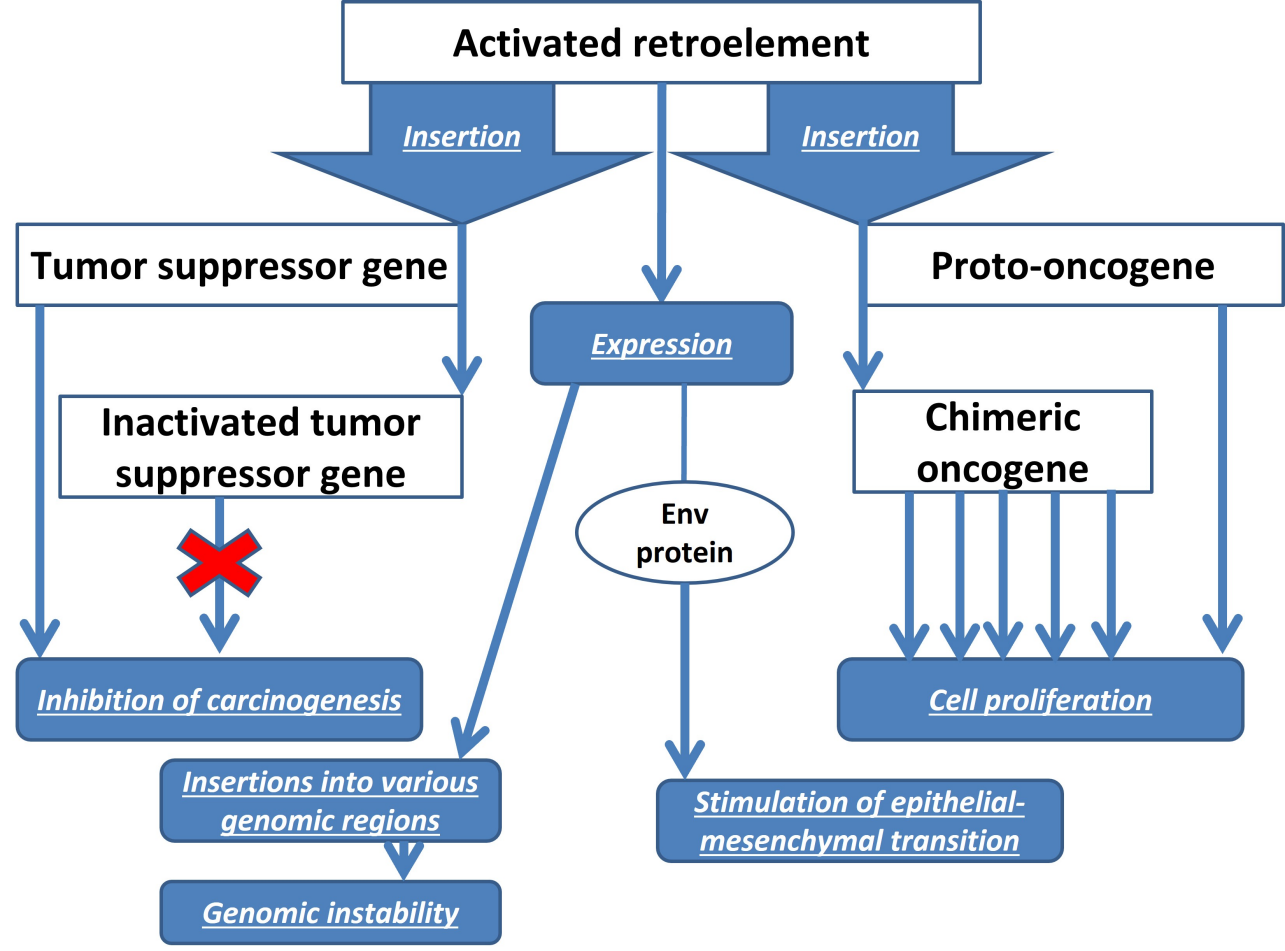
Scheme of the mechanisms of retroelements influence on the development of BC

The envelope protein env of the HERV-K also has oncogenic properties in BC. Stimulation of its expression promotes epithelial-mesenchymal transition of normal breast cells [53]. In the blood of patients with BC, increased levels of the env protein are detected not only of the HERV-K, but also of HERV-H, HERV-P, HERV-R, which decrease under the influence of chemotherapy, to a greater extent than in patients receiving radiation therapy [64]. A pronounced level of HERV-K expression is characteristic of the aggressive basal-like subtype of BC, which suggests the possible use of HERVs as prognostic biomarkers of BC [25]. Similar prospects are planned for antibodies to HERV-K for early diagnosis of BC [65]. Figure 1 shows a diagram of the influence of REs on the development of BC.

In addition to HERV, clinical studies of BC patients have shown significant LINE1 hypomethylation in tumor tissues compared to healthy controls [54]. Moreover, the level of LINE1 hypomethylation in primary BC correlates with a worse prognosis in young patients [66]. The most evolutionarily ancient LINE1 (with a truncated 5’ end and, therefore, inability to transposition) in BC cells exhibit a pronounced ability to bind to transcription factors at their 3’ ends, which may characterize their role in gene regulation in this cancer [24]. Using reverse transcriptase PCR, a negative correlation was identified between LINE1 expression and BC cell differentiation, lymph node involvement, and increased immune infiltration. Moreover, LINE1 has been proposed as an independent biomarker for patients at high risk of TNBC [55].

Since LINE1 enzymes are used for retrotransposition of non-autonomous ERs, activated Alu also plays a role in the development of BC, which contributes to genomic instability, chromosomal deletions and insertions [26]. In relation to invasive BC, Alu and LINE1 hypomethylation correlates with negative ER status, and its extent varies significantly among different BC subtypes [67]. The role of REs as an etiological factor in hereditary BC should be noted. Between 7% and 10% of BC cases are caused by germline heterozygous mutations in the *BRCA1* gene, which contains an insertional mutagenesis hotspot for the 202-bp Alu element. at position c.4475_c.4476 in exon 14 with a variant allele frequency of 22% [68]. In addition, Alu located in the introns of the BRCA1 gene cause non-homologous recombinations between them with the formation of duplications [69].

## Prospects for inhibition of REs in antitumor therapy of BC

The data presented in the previous chapter indicate the important role of HERV, LINE1, and Alu REs in the pathogenesis of BC. Retrotransposition of REs is a factor of genomic instability leading to chromosomal mutations [26] with inactivation of tumor suppressors [70], which is characteristic of BC [63]. Therefore, a rational approach in the treatment of BC is to prevent insertions in order to avoid the formation of cell subclones containing new mutations that contribute to carcinogenesis and chemoresistance. For this purpose, the use of reverse transcriptase inhibitors (RTIs) is promising. The ability of the non-nucleoside RTI efavirenz, which affects LINE1-encoded reverse transcriptase, to suppress proliferation and promote differentiation, has been demonstrated in BC cells. There was repression of genes involved in invasion, migration, and proliferation of BC cells [71]. Similar results were reported in recent studies of efavirenz in TNBC [72]. Nucleoside RTIs stavudine and abacavir promoted apoptosis of BC cells and inhibited their migration ability [73]. In clinical trials survival between women living with HIV and estrogen-receptor positive BC taking efavirenz and nonefavirenz regimens were compared. Results suggesting efavirenz is unlikely driving poorer survival in women living with HIV and estrogen-receptor positive BC [74].

Suppression of REs activity is possible by influencing epigenetic factors. For this purpose, inhibitors of histone demethylases are used, the overexpression of which, leading to stimulation of REs transcription, is characteristic of cancers. In various types of tumors, increased levels of representatives of the KDM5A/B/C/D histone demethylase subfamilies of the JARID1 family are detected, which are demethylated by H3K4me2 and H3K4me3, repressive marks for ER. Histone demethylase inhibitors include CPI-455 and 1,7-naphthyridines, which are effective against chemotherapy-resistant cancers [75]. However, the most promising is the silencing of specific REs involved in BC carcinogenesis, which will make it possible to combine this approach with viral mimicry. Studies of BC have shown the involvement in carcinogenesis of only certain REs (HERV-K) with the absence of the effect of HERV3, HERV-E4-1 [56], which, however, are immunogenic. It indicates the rationality of developing a differentiated approach to viral mimicry aimed at stimulating elements not involved in the development of BC (for example, HERV3, HERV-E4-1) and suppressing pathogenic REs (such as HERV-K). miRNAs can serve as tools for this approach as guides for silencing REs by methylating them in the genome due to the effect of RNA-directed DNA methylation [76]. The complementarity of miRNAs with specific REs is associated with the evolutionary origin of miRNA genes from transposons [58], which may explain the involvement of non-coding RNAs in carcinogenesis.

The basis for differentiated effects on REs involved in BC can be studies of the expression characteristics of REs in different cancers. In particular, a comparison of REs activation in BC, prostate and colon cancers showed locus-specific expression of REs for each tumor type [77]. To determine possible pathways of influence on both miRNAs and transposons involved in the development of BC, I analyzed scientific literature data on the origin of miRNA genes from transposons [58, 78–82] and their role in the development of BC, according to the online resource OncomiR [59]. As a result, I discovered 35 such miRNAs (Table 2). Reduced expression in BC was determined for 14 of these miRNAs*: miR-1249, -1271, -1296, -211, -224, -28, -326, -335, -378a, -487b, -495, -511, -584, and -585. Increased expression in BC was detected for 21 miRNAs* derived from REs: miR-1266, -1269a, -151a, -192, -2114, -2115, -3200, -340, -342, -374a, -374b, -3934, -421, -450b, -493, -545, -551a, -576, -582, -708, and -887. These molecules can be used for targeted therapy of BC. Long non-coding RNAs, the genes of which arose from REs in evolution, can also be used as tools for influencing REs in the treatment of BC. Among them, TROJAN, derived from HERV and expressed in TNBC, should be noted [83].

**Table 2:** Expression changes of transposon-derived miRNAs* in BC [58, 59, 78–82]

**No.**	**LINE-derived miRNA**	**SINE-derived miRNA**	**LTR-retroelement-derived miRNA**	**DNA-transposons-derived miRNA**
1	LINE2/miR-1249 (↓)			
2		MIR/miR-1266 (↑)		
3			ERV-L/miR-1269a (↑)	
4	LINE2/miR-1271 (↓)			
5	LINE2/miR-1296 (↓)			
6	LINE2/miR-151a (↑)			
7	LINE2/miR-192 (↑)			
8	LINE2/miR-211 (↓)			
9	LINE-CR1/miR-2114 (↑)			
10	LINE1/miR-2115 (↑)			
11				MER135/miR-224 (↓)
12	LINE2/miR-28 (↓)			
13			ERV-L/miR-3200 (↑)	
14				hAT-Tip100/miR-326 (↓)
15		MIR/miR-335 (↓)		
16				TcMar/miR-340 (↑)
17		tRNA-RTE/ miR-342 (↑)		
18	LINE2/miR-374a (↑)			
19	LINE2/miR-374b (↑)			
20		MIR/miR-378a (↓)		
21		MIR/miR-3934 (↑)		
22	LINE2/miR-421 (↑)			
23	LINE1/miR-450b (↑)			
24		MIR/miR-487b (↓)		
25	LINE2/miR-493 (↑)			
26			ERV-L/miR-495 (↓)	
27	LINE1/miR-511 (↓)			
28	LINE2/miR-545 (↑)			
29	LINE1/miR-551a (↑)			
30	LINE1/miR-576 (↑)			
31	LINE-CR1/miR-582 (↑)			
32			hAT-Blackjack/miR-584 (↓)	
33			ERV-L/miR-585 (↓)	
34	LINE2/miR-708 (↑)			
35	LINE2/miR-887 (↑)			

↑: increase; ↓: decrease

As can be seen from the Table 2, the pathogenesis of BC involves miRNAs derived from both the most common LINE REs in the human genome (20 miRNAs), as well as from non-autonomous SINE (6 miRNAs) and ERV (4 miRNAs). Since the role of these REs in the pathogenesis of BC has been described [24, 50–55], the use of miRNAs as tools or objects of influence is promising for complex targeted therapy of BC. This is especially important for the method of viral mimicry, in which REs are activated to stimulate an antitumor immune response, potentially being drivers of carcinogenesis, which is a serious problem with this method. Therefore, the use of miRNAs derived from transposable elements that we have identified is one of the possible solutions to this problem, since miRNAs derived from transposable elements can be used to silence REs involved in the pathogenesis of BC [57]. miRNAs are also involved in many other mechanisms of BC development through post-transcriptional regulation of specific genes, as well as interacting with ncRNAs. For example, increased expression of circROBO1 was detected in liver metastases of BC, which promotes tumor growth and metastasis to the liver. Knockdown of circROBO1 inhibited the proliferation, migration, and invasion of BC cells. This circRNA activates *KLF5* by sponging miR-217-5p. Due to this, circROBO1 inhibited selective autophagy. *KLF5* is a direct target of miR-217-5p and activates the mTOR-PI3K-ATK. Therefore, miR-217-5p exhibits tumor suppressor properties and can be used in the complex therapy of metastatic BC [84].

It was also found that isoliquiritigenin has a powerful anticancer effect on the proliferation of BC cells and promotes an increase miR-200c level by demethylating the promoter region of the gene. TNBC tissues are characterized by low expression of miR-200c, the target of which is the mRNA of the c-Jun gene (a protein product that promotes proliferation, cell survival, and carcinogenesis). Under the influence of isoliquiritegenin, increased levels of miR-200c inhibit epithelial-mesenchymal transition [85]. Thus, miRNAs can mediate the effects of a number of anticancer drugs, suggesting the potential of targeted therapy using miRNAs as tools. miR-637 inhibits *STAT3* gene expression, promoting brain metastasis in TNBC. CircKIF4A also binds complementarily to the 3’-UTR of the mRNA. STAT3 can regulate autophagy by regulating the transcription of autophagy-related genes such as *Beclin*, *PIK3C3* and *CTSB*. Autophagy dysfunction influences cell fate in a significant way and may play a fundamental role in BC progression [86].

In a similar way, the miRNAs shown in the Table 2, derived from transposable elements, interact with other ncRNAs in the regulation of BC carcinogenesis. For example, LINE2-derived miR-1271 [58, 78–82], which is a tumor suppressor, is a target of circ-ABCB10, which promotes BC proliferation and progression [87]. miR-1249, derived from LINE2 [49, 68–72], inhibits the proliferation and migration of BC cells by affecting the HOXB8 axis and interacts with lncRNA MIF-AS1, which is a sponge for this miRNA [88]. Therefore, one of the ways to activate such tumor suppressor miRNAs may be the use of antisense oligonucleotides that inhibit lncRNAs [89] interacting with miRNAs. It is also possible to use miRNAs and their mimetics in complex therapy for BC. A detailed analysis of transposable elements involved in BC carcinogenesis will allow us to identify REs that do not affect BC pathogenesis and that are capable of activating antitumor immunity in the method of viral mimicry for BC treatment. For these purposes, specific antisense oligonucleotides can be used to suppress miRNAs involved in the inhibition of such specific REs. The candidates are those presented in the Table 2, derived from transposable elements miRNAs, which expression is increased in BC.

## Conclusions

**Figure 2:**
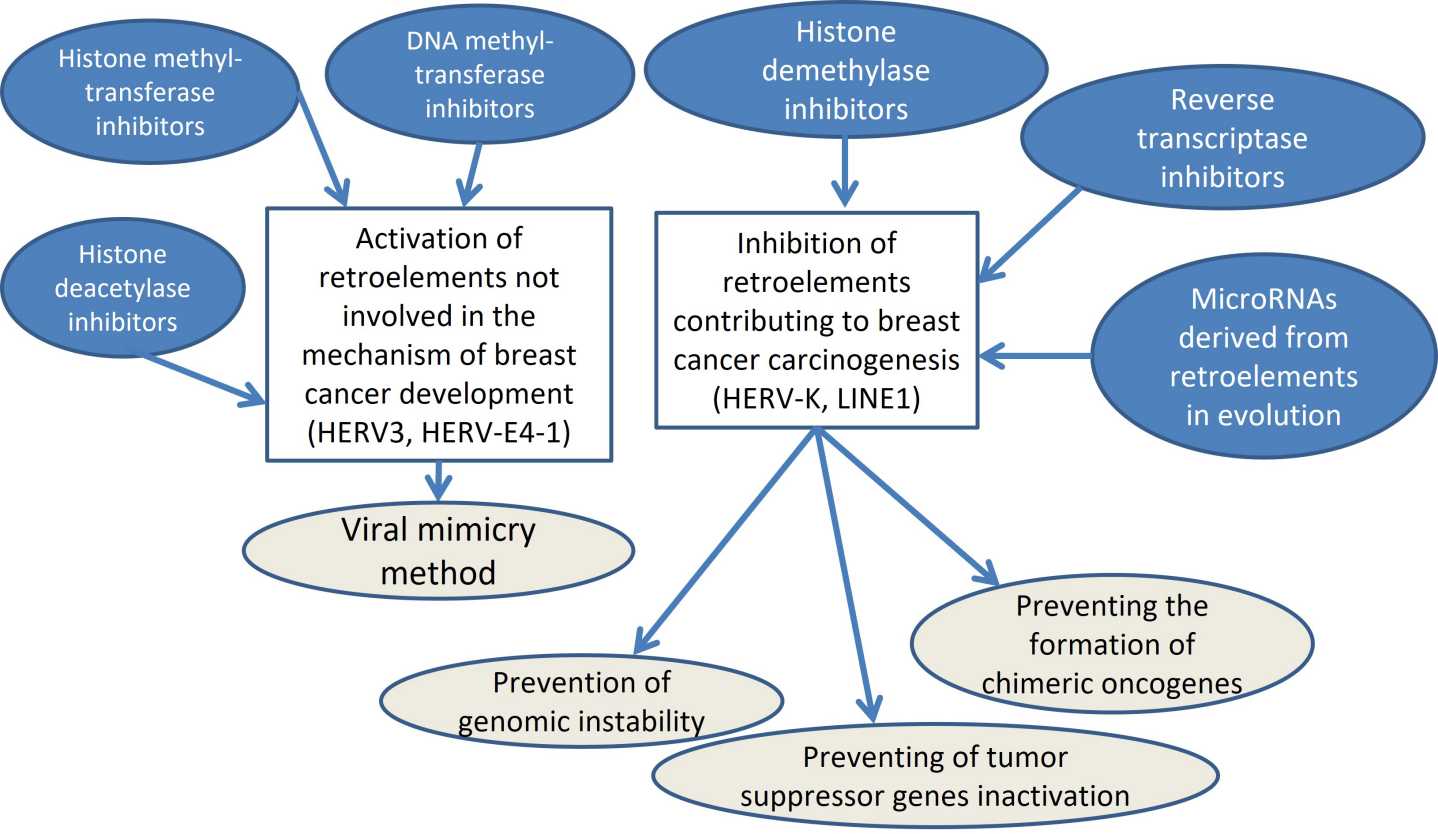
Scheme of complex proposed interventions in BC treatment

One of the promising areas of BC immunotherapy may be the method of viral mimicry, since anti-PD-1/PD-L1 turned out to be ineffective even for the most immunogenic TNBC. Experimental studies on BC cell lines and laboratory animals have shown the potential of using inhibitors of DNA methyltransferases, histone deacetylases and histone methyltransferases due to the activation of the interferon response by double-stranded transcripts of epigenetically stimulated REs. The additional effect is due to the elimination of repression marks in the promoter regions of tumor suppressor genes. However, in addition to the beneficial effects regarding BC carcinogenesis, the “viral mimicry” method has challenges since a number of scientific articles have proven the role of activation of HERVs, LINE1s, and Alus in the carcinogenesis of BC, which indicates a possible stimulation of BC growth under the influence of inhibitors of DNA methyltransferases, histone deacetylases and histone methyltransferases. The possible solutions in the field are the inhibition of transposable elements involved in BC carcinogenesis, while simultaneously stimulating REs that are not involved in tumor development and stimulate the antitumor response. Specific miRNAs that originate from transposable elements in evolution and inhibit their expression can be used as tools for this approach. This article presents the results of the discovery of 35 miRNAs derived from transposable elements, which can be used in the future for such an integrated approach in targeted therapy for BC. Moreover, a comprehensive approach to using these drugs together with RTIs and specific inhibition of REs genes under the influence of their complementary miRNAs is rational (Figure 2).

## Abbreviations

BC: breast cancer
CTLA4: cytotoxic T-lymphocyte protein 4
env: envelope
ER: estrogen receptors
ERVs: endogenous retroviruses
HERV: human endogenous retroviruses
LINE: long interspersed nuclear element
miRNAs: microRNAs
non-LTR: non-long terminal repeat
PD-1: programmed cell death 1
PD-L1: programmed cell death ligand 1
PR: progesterone receptors
REs: retroelements
RTIs: reverse transcriptase inhibitors
SINE: short interspersed nuclear element
SVA: SINE-VNTR-Alu retroelement
TNBC: triple-negative BC

## Declarations

### Copyright

© The Author(s) 2024.
